# Using Personal Mobile Phones to Assess Dietary Intake in Free-Living Adolescents: Comparison of Face-to-Face Versus Telephone Training

**DOI:** 10.2196/mhealth.5418

**Published:** 2016-07-29

**Authors:** Gina Segovia-Siapco, Joan Sabaté

**Affiliations:** ^1^ Center for Nutrition, Healthy Lifestyle, and Disease Prevention School of Public Health Loma Linda University Loma Linda, CA United States

**Keywords:** adolescents, dietary assessment, dietary records, digital images, follow-up, mobile phones, real-time support, technology

## Abstract

**Background:**

Traditional paper-based methods to assess food intake can be cumbersome for adolescents; use of mobile phones to track and photograph what they eat may be a more convenient, reliable, and compelling way to collect data.

**Objective:**

Our aims were to determine (1) the feasibility of using personal mobile phones to send food records with digital images (FRDIs) among free-living adolescents and (2) whether the quality of food records differed between a high-level intervention group (ie, face-to-face training plus real-time support) and a low-level intervention group (ie, telephone training plus next-day follow-up).

**Methods:**

Adolescents (N=42, 11 males and 31 females) aged 12-18 years who had a mobile phone with camera enrolled in the study via consecutive sampling. The first group (n=21) received face-to-face training while the second group (n=21) was trained via telephone. Participants received a fiducial marker (FM) and completed a 1-day FRDI using their mobile phones. At every eating occasion, participants were to (1) take clear images of their meals/food with a correctly placed fiducial marker before eating, (2) send the image immediately to a designated email address, (3) right after completing a meal, send a text message listing the time and name of the meal, foods eaten, and amounts eaten, and (4) before sleep, send an “end” text message to indicate completion of food recording. Those who received face-to-face training received real-time support during reporting; those trained by telephone received next-day follow-up. Descriptive statistics and comparison tests were used to determine performance of the groups.

**Results:**

All participants (N=42) who underwent training completed their 1-day FRDI. A significantly greater proportion of the low-level intervention group compared to the high-level intervention group placed their FM correctly in the image (95% vs 43%, *P*<.001), had complete information for each meal in their food record (95% vs 71%, *P*=.04), and had a higher overall score in meeting the criteria for food recording (4.3 vs 3.4 out of 5 points). Both groups had energy intake values that moderately correlated with their estimated energy requirements: low-intervention r=.55; high-intervention r=.51.

**Conclusions:**

Using personal mobile phones to report dietary intake via texting and digital images is feasible among free-living adolescents. Real-time support or high-level intervention does not guarantee better food recording quality among adolescents.

## Introduction

Dietary assessment is an important component in the investigation of diet-health relationships; thus, it is crucial that methods used to determine intake are reliable and valid. Several approaches are used to measure what people eat, but each has inherent biases and limitations. Specific characteristics of populations also contribute to bias in assessing dietary intake. For instance, underreporting is common among obese and overweight individuals [1-3]. Inaccuracy in dietary assessment is compounded further by an exponential increase of food products on the market, a glut that necessitates timely upkeep of existing food and nutrient databases to prevent them from getting outdated.

An accurate assessment of dietary consumption by adolescents is particularly challenging. Youth in this age group have difficulty conceptualizing or averaging food portion sizes [4], have a limited food vocabulary, and lack the patience and perseverance to engage in food recording [5,6]. Recall of routine or regularly occurring events can be relatively accurate, but adolescents tend to skip meals [7,8] and have wide and inconsistent day-to-day variation in the frequency, composition, and timing of their meals [6]; thus, their recall could be unreliable unless they record what they eat immediately after meals and snacks.

Often, social pressure or the lack of confidentiality also makes adolescents unwilling to undertake paper-based food recording; in addition, they tend to modify their intake to ease the burden associated with food recording [9]. Although conventional methods of dietary assessment can be cumbersome for adolescents, innovations that tap into their technological skills may keep them engaged in reporting their dietary intake [5]. Given that the traditional paper-and-pen food recording entails detailed descriptions of foods eaten, pictures taken during eating events can potentially reduce such burden because pictures can convey those details as well.

Mobile electronic devices have made their way into most households—approximately 78% of adolescents own mobile phones, 23% own a tablet computer [10], and a large percentage are constantly occupied with technological devices (eg, computers and mobile phones) [11]. Mobile electronics are already part of the adolescent lifestyle. Digital photography used as either the main method to record intake or in conjunction with the traditional food record can provide objective information about dietary intake, reduce participant burden, and potentially decrease the need for portion size estimation [12-15]. Since digital images can simplify food recording [16], use of the camera feature of mobile phones had been tested among adolescents in both monitored [17-19] and free-living [20] settings.

While considerable work has been done in dietary assessment methods for the purpose of reducing participant and research burden, most of the work has not been completed and is still undergoing refinement or validation. Development of software and mobile phone apps for dietary assessment purposes can be exceedingly costly, extremely challenging, and may take several years to complete [21]. However, existing mobile phone technology (eg, camera and short message service [SMS] text messaging) may be efficiently utilized to serve practically the same purpose in collecting dietary information until apps for mobile phones are made available for researchers at a reasonable cost. Adolescents were willing to use SMS text messaging when reporting their health information needs [22]. Likewise, the use of SMS text messaging and camera features of mobile phones needs to be tried when assessing dietary intake of adolescents.

We determined the feasibility of assessing the dietary intake of free-living adolescents by using their own mobile phones to do digital image-assisted food recording. We also explored whether the quality of food records from these adolescents would differ based on the type of training and support provided during food recording. It had been suggested that automated feedback may improve intake reporting accuracy of adolescents [17,20,23]. Considering that the concept of automated feedback could be mimicked with real-time human monitoring, we used the latter as a proxy to test whether real-time feedback would indeed improve recording accuracy. We hypothesized that high-level intervention, which includes face-to-face training and real-time support, would promote higher-quality food records compared to low-level intervention, which includes training through telephone conversation and next-day follow-up.

## Methods

### Participant Selection and Study Design

We recruited 12- to 18-year-old adolescents from select middle and high schools to be part of a study to determine the feasibility of using personal mobile phones in keeping food records with digital images (FRDIs). Participants were recruited via consecutive sampling in April and August 2012. Eligibility requirements included ownership of a mobile phone with a camera, unlimited texting/calling, and the ability to send SMS text messages and images to an email address. All study protocols were approved by the Loma Linda University Institutional Review Board. We obtained consent of parents and their children’s assent prior to starting the study.

To avoid cross-contamination and for logistical reasons, this pilot study was designed such that the first group of available volunteers who met the eligibility criteria were assigned to high-level intervention while the second group who joined the study later were assigned to low-level intervention. High-level intervention was conducted in May 2012, and low-level intervention in September 2012. Participants of the high-level intervention (n=21) were instructed to watch an instructional video prior to an in-depth, one-on-one, face-to-face training session, which took place in the school. Three research assistants were assigned to do the face-to-face training. On the other hand, personal training for the participants of the low-level intervention (n=21) was delivered via a one-one-one telephone conversation by a research assistant. Each participant received illustrated instructions beforehand, which served as the training material during the telephone conversation. Instructional content was similar for both groups. After training, participants were scheduled to perform a 1-day FRDI. All the participants who joined the study (N=42) completed the training, did their 1-day FRDI, and received a US $10 iTunes gift card as incentive.

### Protocol for Food Recording With Digital Images

**Figure 1:**
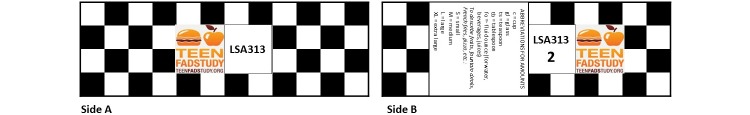
Fiducial marker with two sides that show the identification (ID) number of the participant and the study logo (Sides A and B), and commonly used portion size measurements (Side B).

Each participant received a fiducial marker (FM)—an object of known dimension from which sizes of nearby objects can be determined—which was a two-sided, 6-inch x 2-inch piece of laminated cardboard, checkered with 0.5-inch black and white squares. Placement of the FM in the image allowed us to approximate the size of objects (eg, eating utensils and foods). The FM had the identification number (ID) of the participant and the study logo on one side (see Figure 1, Side A); on the other side, it had the same identification number with the number “2” and a list of common food measurements (see Figure 1, Side B). These were sent to school administrative offices for distribution to study participants. The participant ID on the marker enabled matching and proper identification of received images. It also ensured that the images were not fabrications (eg, food images from the Internet). The schools allowed participants to use their mobile phones for the study at designated places and at break/lunch times.

We set up a secure email account for the study; it served as the interface between participants and the research team. We created a specific inbox for each participant and used filters to direct incoming mail to the appropriate inboxes. Both groups were trained to send food records as SMS text messages with images of their meals to the study email address. To make sure they could do so, they were required to respond to an instructional SMS text message from the research team. It asked them to send a return message and an image of their FM to the specified email address. Participants were also told to save the email address in their contact list to facilitate correspondence with the research team. The day before filing their 1-day FRDI, each participant received an email reminder to charge his or her mobile phone, bring the FM to school the following day, and review the illustrative instructions one more time.

**Figure 2:**
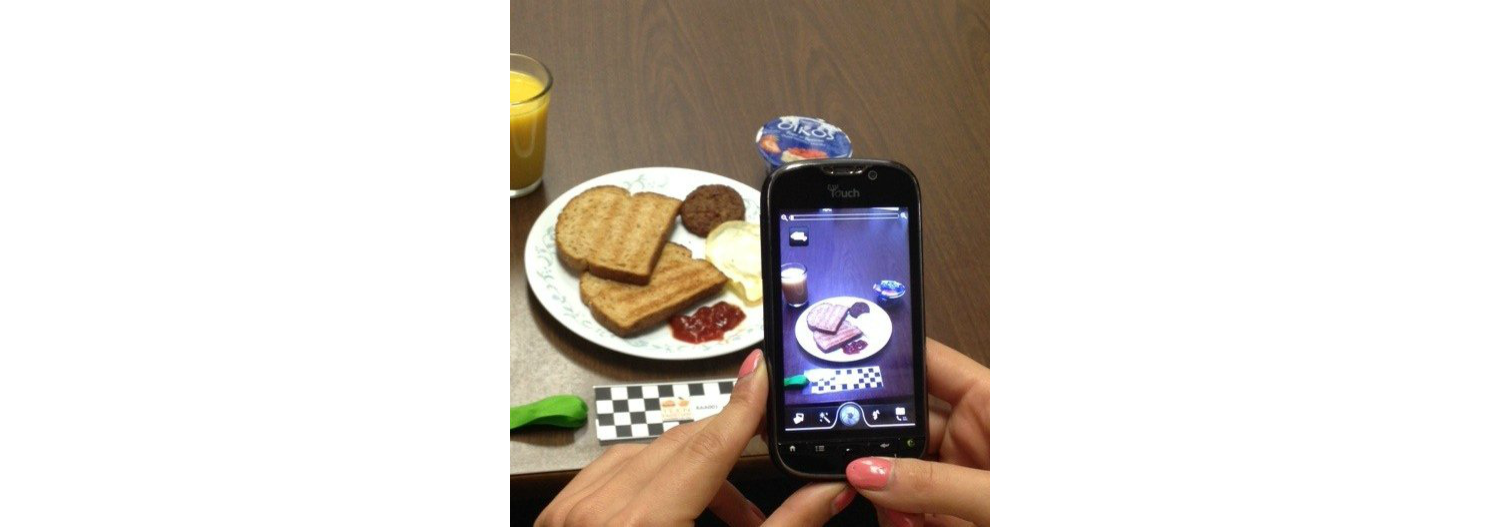
Image of the food setting with the fiducial marker (FM). The participant captures the entire food setting at one step away from the edge of the table. The height of the mobile phone camera above the setting (~1 foot) is first determined using the ribbon on the FM. When stepping back, elbows are clipped to one's sides to maintain position of the arms. Keeping the arms steady, the mobile phone camera is tilted up or down until the whole setting can be seen on the screen.

**Figure 3:**
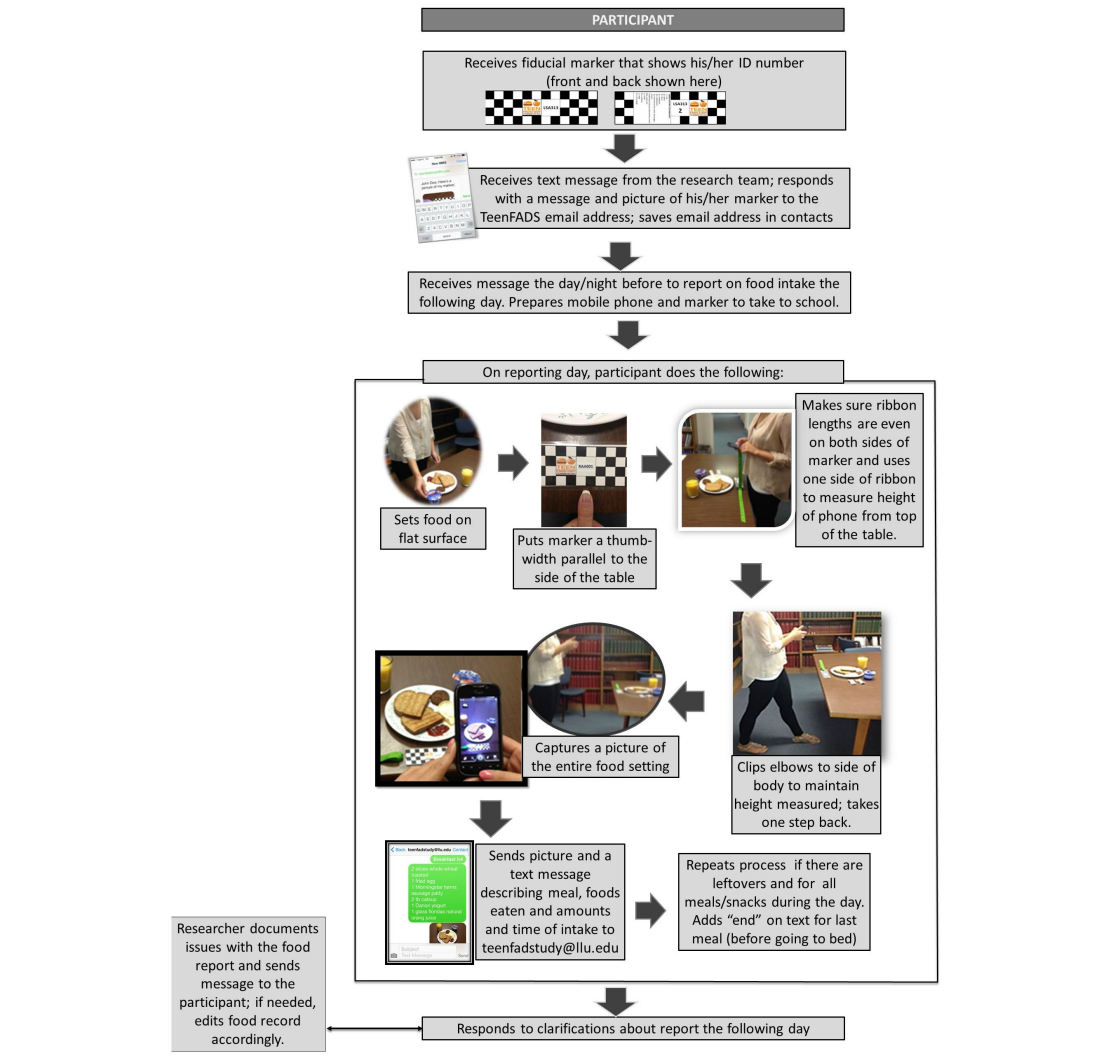
Diagram of the food record with digital images (FRDI) using personal mobile phones by adolescents. High-level intervention (ie, real-time support) participant responds to prompts and feedback by researcher; low-level intervention participant responds to researcher feedback after reporting day (ie, next-day) follow-up. ID: identification.

At every eating occasion, participants took an image of the food setting with the FM placed below the setting and parallel to the table’s edge (see Figure 2). Once finished eating, they sent the image with an SMS text message that listed the meal name, time (ie, since not all images would necessarily include metadata on the time and/or date the image was taken), foods, and corresponding amounts eaten. They repeated the process for second helpings and/or additional beverages and foods. They were required to send an after-meal image if there were leftovers. Otherwise, they could just send an SMS text message indicating that they had eaten everything in the original image. Before retiring for the night, they texted a closure message with the time and the term “end” to indicate that nothing more would be consumed after that time. Figure 3 shows a schematic diagram of this process.

### Collection of Data: High-Level Versus Low-Level Intervention

Research assistants were trained in providing both real-time support and follow-up to ensure uniformity in following protocols. Both groups were sent a reminder message the day before their scheduled reporting day to ready their mobile phones and bring their FM to school the following day.

High-level intervention, characterized by real-time support, involved active synchronous monitoring. During the scheduled reporting day, trained research assistants monitored the high-level intervention participants from 5:30 AM to 10:00 PM. Real-time support entailed sending reminder messages or prompts, particularly when SMS text messages and images were not coming in around expected eating times, and on-the-spot instructions when they saw reporting errors (eg, blurred or missing FM). The monitors also reminded participants to send the closure “end” messages before they retired for the night.

While actively monitoring the high-level intervention participants, texted images and corresponding messages from the email inboxes were collated into PowerPoint slides. Each slide included an image and text report of a meal or food/beverage; Figure 3 shows this process. In addition, researchers logged observations and issues encountered during monitoring.

**Figure 4:**
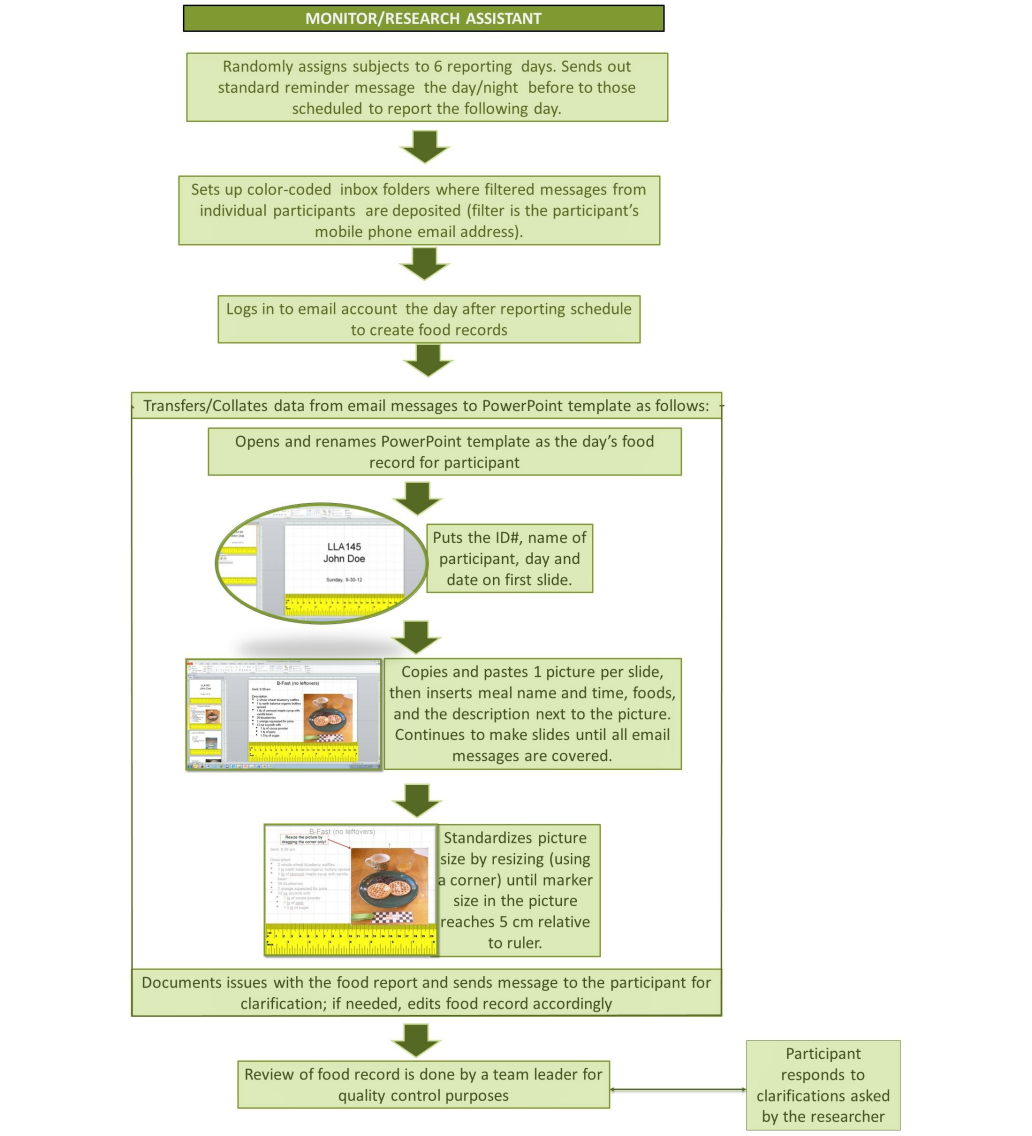
Diagram of the researcher procedure in collecting food records with digital images (FRDIs) using mobile phones by adolescents. The researcher provides real-time prompts and feedback during high-level intervention, but only asks for clarifications on reports from low-level intervention participants during next-day follow-up. ID: identification.

Low-level intervention only entailed next-day follow-up. No reminders or any kind of monitoring was given during the reporting day of low-level intervention participants. The following day, research assistants checked the participants’ reports and transferred the images and corresponding SMS text messages from their email inboxes to their individual food records (ie, PowerPoint slides). Observations about the messages and images were logged. Participants were informed about items that needed clarification, and their responses were entered into their PowerPoint record (see Figure 4). For both high-level and low-level interventions, the food record was a product of the participant’s (text and image) and the research assistant’s (interpretation and assembly of sent texts and images) contributions.

### Data Analysis

To determine the quality of reports, we computed the proportion of participants in each group who (1) sent images with their FM, (2) correctly placed their FM relative to the food arrangement, (3) sent an accompanying SMS text message that described the foods eaten and/or if there were any leftovers, (4) gave complete information, including the time, meal name, food list, and corresponding amounts eaten, and (5) sent good-quality images (ie, images with foods that could be clearly identified).

We also created a scoring system to determine the quality of reports for each individual based on the following criteria: (1) proper placement of the FM in all images (1 point), (2) complete meal information—with time of intake, name of meal, and the names and corresponding amounts of foods eaten—in the texted food report (1 point), (3) texting the closure “end” message (1 point), (4) each image accompanied by a text message (1 point), and (5) quality of images (0 point if all images were blurred, 0.5 point if some were blurred, or 1 point if all were of good quality). Since participants were instructed to take an *after* image only if there were leftovers, no score was assigned to *before-and-after* photos. We used the quality of reports to determine adherence to instructions. The maximum score was 5 points, which was equivalent to 100% adherence to instructions.

Descriptive statistical (ie, frequencies) and comparison (ie, paired and independent *t* tests) analyses were used to evaluate the quality of FRDIs. All analyses were done using IBM SPSS version 22.0 for Windows (IBM Corp, Armonk, NY) [24].

To determine if type of training would differentiate reported intake among these adolescents, we compared their energy intake from the 1-day FRDI with their estimated energy requirement (EER). Energy intake (EI) from food records was determined by using the Nutrition Data Systems for Research version 2012 software developed by the Nutrition Coordinating Center, University of Minnesota, Minneapolis, MN. EER was computed using the equations to calculate the Dietary Reference Intake for energy [25]; physical activity level (PAL) was set at low active (PAL of 1.40-1.59), which has equivalent physical activity (PA) values of 1.13 for boys and 1.16 for girls:

EER_boys_,_9-18 years old_ = 88.5-(61.9 x age [y]) + PA x {(26.7 x weight [kg]) + (903 x height [m])} + 25 (1)

EER_girls_,_9-18 years old_ = 135.3-(30.8 x age [y]) + PA x {(10.0 x weight [kg]) + (934 x height [m])} + 25 (2)

Accuracy of energy reporting was determined using the EI:EER ratio. Given that we have a small sample size, the cutoff points determined from 1-day dietary information on a more representative sample of adolescents—the National Health and Nutrition Examination Survey (NHANES) 2003-2011 data (N=14,044 children and adolescents) [26]—were applied to this study. The cutoff points to categorize under-, plausible, and overreporters were <0.61, 0.61-1.64, and >1.64, respectively.

## Results

### Profile of Participants

The low-level intervention group was composed of 15 females and 6 males, while the high-level intervention group had 16 females and 5 males of similar age. Table 1 describes the composition and demographic characteristics of the participants as a whole and by groups. There were no significant differences in gender, age, or ethnicity. The majority of students were 16 years of age or older, and there were more non-Hispanic whites in the low-level compared with the high-level intervention group. Average body mass index (BMI) *Z* scores and BMI percentiles for both groups were within the normal category.

### Assessment of 1-Day Food Reports by Group

The number of meals reported ranged from 2 to 6, with the majority of participants reporting 3 meals (9/21, 43% high-level intervention group; 11/21, 52% low-level intervention group). Participants sent images for every eating occasion and each additional serving, as well as *after-eating* images when there were leftovers. The number of images showing the FM ranged from 1 to 11 for the high-level intervention group and from 1 to 6 for the low-level intervention group. *Before-eating* images ranged from 2 to 8, with a mean of 4.1 (SD 1.5) for the high-level intervention group and 3.8 (SD 1.5) for the low-level intervention group, but not all of these images included the FM. Since participants were instructed to only send *after-eating* images if there were leftovers, they sent very few. The majority of participants from both high-level (15/21, 71%, mean 0.5 [SD 1.2]) and low-level (17/21, 81%, mean 0.2 [SD 0.5]) intervention groups did not send *after-eating* images. Out of the 21 participants in the low-level intervention, 4 (19%) had no problems with their reports and, thus, did not need follow-up; 14 (67%) responded in a timely manner to follow-up; and 3 (14%) either did not respond or had a delayed response.

**Table 1:** Demographic profile of participants.

Demographic characteristics		All participants (N=42), n (%) or mean (SD)	High-level intervention^a^ (n=21), n (%) or mean (SD)	Low-level intervention^b^ (n=21), n (%) or mean (SD)
**Gender, n (%)**				
	Male	11 (26)	5 (24)	6 (29)
	Female	31 (74)	16 (76)	15 (71)
**Ethnicity, n (%)**				
	Non-Hispanic white	13 (31)	4 (19)	9 (43)
	Other ethnicities	29 (69)	17 (81)	12 (57)
Age in years, mean (SD)		15.8 (1.9)	15.9 (2.1)	15.6 (1.6)
**Age group, n (%)**				
	12-13 years	7 (17)	5 (23)	2 (10)
	14-15 years	9 (21)	2 (10)	7 (33)
	16-18 years	26 (62)	14 (67)	12 (57)
BMI^c^*Z* score, mean (SD)		0.50 (0.89)	0.46 (0.80)	0.54 (0.99)
BMI percentile, mean (SD)		64.3 (25.9)	63.9 (23.6)	64.7 (28.6)

^a^High-level intervention involved one-on-one, face-to-face training and real-time support during food recording day.

^b^Low-level intervention involved one-on-one training via telephone conversation with only a follow-up after the food recording day.

^c^BMI: body mass index.

Table 2 shows the assessment of the 1-day FRDIs for the two groups. The total number of *meals eaten* images with the FM were similar for the two groups. However, the low-level intervention group sent more images with correctly placed FMs compared with the high-level intervention group (mean 4.0 [SD 1.7] vs mean 2.1 [SD 2.4], respectively). Compared with their high-level intervention peers, more participants in the low-level intervention group had their FM in all the images they sent (20/21, 95% vs 15/21, 71%, respectively). These FMs were correctly placed relative to their food settings more often by the low-level versus high-level intervention group (20/21, 95% vs 9/21, 43%, respectively). SMS text messages sent with accompanying images from those in the high-level intervention group had more missing information compared with participants in the low-level intervention group. The majority of participants from the high-level intervention group had missing information on their texted report, such as time and name of meal (12/21, 57%); 1 did not include amounts of foods eaten and another reported a food in the text that was not in the image. In the low-level intervention group, 2 out of 21 (10%) did not give the meal time and name, and 1 out of 21 (5%) did not have amounts of foods eaten. In the high-level intervention group, a total of 5 participants out of 21 (24%) did not complete their FRDI: 3 did not report dinner, 1 did not report breakfast, and 1 did not report lunch. Out of 21 low-level intervention participants, 1 (5%) did not report lunch.

**Table 2:** Assessment of the 1-day food record with digital images, by group.

Factor		High-Level intervention^a^ (n=21)		Low-Level intervention^b^ (n=21)	
		Count	Mean (SD)	Count	Mean (SD)
**Total number of:**					
	Meals eaten	75	3.6 (1.3)	73	3.5 (1.1)
	Images with FM^c^	80	3.8 (1.3)	84	4.0 (1.7)
	Images with correctly placed FM	45	2.1 (2.4)	83	4.0 (1.7)
	Images accompanied by text report^d^	95	4.5 (2.3)	85	4.1 (1.7)
	Text reports^e^ with missing information	30	1.4 (1.1)	5	0.2 (0.6)
**Number of participants with:**					
	Completed FRDI^f^	15	N/A^g^	20	N/A
	“End” message	7	N/A	8	N/A
	FM in all images	15	N/A	20	N/A
	Correctly placed FM in images	9	N/A	20	N/A

^a^High-level intervention involved one-on-one, face-to-face training and real-time support during food recording day.

^b^Low-level intervention involved one-on-one training via telephone with only a follow-up after the food recording day.

^c^FM: fiducial marker.

^d^Image—showing or not showing fiducial marker—accompanied by the food report text.

^e^Text information should include meal name—breakfast, snack, lunch, or dinner/supper— meal time, and foods and corresponding amounts eaten.

^f^FRDI: food record with digital images. Completed FRDI refers to reporting all meals eaten and reporting not eating a main meal during food recording.

^g^N/A: not applicable.

### Quality of 1-Day Food Record and Energy Intake Reports

Table 3 shows the quality of the 1-day FRDIs submitted by the participants based on the criteria used to determine compliance with the requirements for food recording. Overall, a greater percentage of participants in the low-level intervention complied with the requirements and had a higher total score for meeting the criteria compared with those in high-level intervention.

**Table 3:** Proportion of adolescents that met the requirements for food recording with digital images, by group.

Criteria^a^	High-Level intervention^b^ (n=21)	Low-Level intervention^c^ (n=21)	*P* ^d^
1. Correctly placed FM^e^ in the image, n (%)	9 (43)	20 (95)	<.001
2. Only good-quality images for whole report, n (%)	15 (71)	20 (95)	.11
3. Texted food intake accompanied by image with FM, n (%)	17 (81)	20 (95)	.15
4. Complete information for each meal in FRDI^f^, n (%)	15 (71)	20 (95)	.04
5. Sent “end” message, n (%)	9 (33)	8 (38)	.75
Total score for meeting criteria, mean (SD)	3.4 (1.1)	4.3 (0.7)	.01

^a^Each criterion met was worth 1 point, except for image quality, where a score of 1 was given if all images in the food record with digital images (FRDI) were of good quality, 0.5 if at least half were of good quality, and 0 if less than half were of good quality.

^b^High-level intervention involved one-on-one, face-to-face training and real-time support during food recording day.

^c^Low-level intervention involved one-on-one training via telephone with only a follow-up after the food recording day.

^d^Chi-square test for each criterion and Mann-Whitney U test for the total score.

^e^FM: fiducial marker.

^f^FRDI: food record with digital images. Information on meal name—breakfast, snack, lunch, or dinner/supper—meal time, and foods and corresponding amounts eaten were included in text messages.

**Table 4:** Comparison of reported energy intake (EI)^a^ with estimated energy requirement (EER)^b^ according to type of intervention.

Intervention group	Correlation (*r*)^c^	*P*	EI (kcal), median (IQR^d^)	EER (kcal), median (IQR)	Energy intake report^e^ (n=21), n (%)		
					Under	Plausible	Over
High-level group	.51	.02	1526 (1214)	1808 (336)	7 (33)	14 (67)	0 (0)
Low-level group	.55	.01	1655 (1275)	1837 (679)	7 (33)	14 (67)	0 (0)

^a^EI: energy intake reported on the 1-day food record with digital images.

^b^EER: estimated energy requirement based on Dietary Reference Intake equations (see Data Analysis section) to estimate energy requirement for boys and girls, 9-18 years old. Physical activity level (PAL) was set at low active (PAL of 1.40-1.59, equivalent to physical activity [PA]=1.13 for boys and PA=1.16 for girls) for lack of physical activity data.

^c^Spearman rank correlation coefficient (*r*) between EI and EER.

^d^IQR: interquartile range.

^e^Energy intake is underreported if EI:EER<0.61, plausibly reported if EI:EER is 0.61-1.64, and overreported if EI:EER>1.64.

Comparison between the EI and EER for both groups are shown in Table 4. Energy intake reports on the FRDI and correlations between EI and EER were similar between the two groups. The counts of energy intake reports considered *plausible* and *underreported* based on the cutoff points adopted from Mukarami and Livingstone [26] were exactly the same in both groups.

## Discussion

### Principal Findings

This study demonstrated that it is feasible to assess dietary intake in free-living adolescents, who were willing to use their own mobile phones, in taking digital photographs and texting their food intake according to the training they received. Our study also showed that low-level intervention (ie, training via telephone conversation plus next-day follow-up) yielded higher-quality reports compared to high-level intervention (ie, face-to-face training plus real-time support during food recording).

Instructions on how to take images, place the fiducial markers, text the details of their food intake, and send the information to the email address we set up solely for the study were followed carefully by the majority of the participants. Adherence to instructions in this study of free-living adolescents was higher compared with that in a study that determined how amenable adolescents were to recording their dietary intake using a mobile phone app [20]. An age difference might explain this disparity—our subjects were older (ie, 12-18 years old) than those in the previous study (ie, 11-14 years old). In-depth, one-on-one training and the short duration of the study also possibly contributed to better compliance. We found that this innovative approach of utilizing existing resources (ie, asking participants to use their own mobile phones) in reporting dietary intake is not only attainable, but could also potentially save financial resources (eg, cost of phone service fees and researcher-provided mobile phones for participants). Conducting the study in a free-living situation and during school days, which are relevant routines for school-age youth, also renders good external validity. This study also allowed us to examine if real-time feedback, albeit in a different delivery mode than automated feedback suggested by investigators [17,20,23], is an appropriate method to improve intake reporting accuracy among adolescents.

Our results indicate that it does not take a high-level intervention to get better-quality 1-day FRDIs from adolescents. Compared to the high-level intervention participants, the low-level intervention group complied better with the requirements of food recording; all of them had their FM during the day of reporting as evidenced by the appearance of FMs in all images, and a higher proportion of this group also provided images with correctly placed FMs—an important consideration when estimating portion sizes—good quality images, SMS text message image with FM pairs, and food records with complete information. The low-level intervention group also met more quality criteria and had higher total scores, indicating greater adherence to instructions. A possible explanation for this difference could be the reliance of the low-level intervention group on the pictorial instructions, which many in the group brought with them to school during their reporting day. Extra emphasis was given to reviewing the pictorial instructions in the food recording reminder message sent the day before to the low-level intervention group. On the other hand, the high-level intervention group was simply reminded about food recording the following day. Both groups, however, were reminded to prepare their phones and FM; despite that, two of the high-level intervention members forgot to bring their FMs to school and, thus, the FM was missing in their images. Since we assumed that participants read their reminder messages, we did not verify if the reminders were actually read.

The reported energy intake on the FRDI and proportion of underreported and plausibly reported energy intakes were similar for both groups. The agreement between the EI and EER was moderate, which indicates that the use of mobile phones in recording dietary intake assisted by digital images provided good estimates of a day’s intake. However, energy intake based on just one day would be insufficient to determine over- or underreporting given the wide day-to-day intake variation especially among adolescents [27]. Also, there is evidence that adolescents tend to underreport their intake, which becomes more pronounced as weight increases [28,29]. Since some of our participants skipped a meal during the day (6/42, 14%), this could partly explain why median intakes of energy for both groups were lower compared to the EER.

Contrary to our hypothesis, real-time support during food recording did not produce better outcomes. Logs during real-time monitoring consistently showed nonresponse among participants who were asked to take better images with properly placed FMs. Since we collected the 1-day FRDIs during school days, schoolwork and activities and short break times might have accounted for the nonresponse or tendency of the face-to-face participants to ignore text reminders from researchers. Although we asked school administrators to exempt study participants from the rule of not using their mobile phones while at school, classroom teachers may have enforced the rule in their classes without exception. In addition, although we did not find any issues when we pretested our email interface on different mobile phones and service carriers, delays in communication exchanges during the monitoring may have played a role as well. In another study, poor compliance was observed when adolescents were preoccupied with a more engaging activity (ie, a minor league baseball game) [23]. Given the results of our human-mediated prompts and reminders, further investigation on how automated prompts/reminders compare to human-mediated intervention in facilitating accuracy in reporting diet among adolescents would be needed.

Better results from low-level versus high-level intervention have implications for the design of subsequent studies. Manually administered real-time support or immediate feedback was more time-consuming than next-day follow-up. To anticipate the volume of incoming messages based on the number of scheduled participants, we needed monitors on hand from the approximate times for breakfast (ie, from 5:30 AM) until late-night snacks (ie, approximately 10:00 PM). To make immediate feedback cost-effective, a proper balance between manpower needs and peak demand was necessary. On the other hand, a next-day follow-up intervention allowed time to identify issues with full reports, ask for clarifications, and then make changes as needed. We scheduled follow-up at mutually agreed-upon times, ensuring that the participant would be available when contacted. This arrangement eliminated the need for excess manpower and was, thus, more economical. Monitor time was also well-spent in collating text messages and images into PowerPoint slides. Although this process was also time-consuming, we found such follow-up to be a crucial step in ascertaining the accuracy of collected data.

Food record apps for mobile phones lessen the burden associated with the traditional paper-and-pen food recording, since subjects are required to only take quality images and to confirm or adjust their input based on a feedback loop after the automated identification and quantification of food images [18]. The concept we used in our method is similar: take good-quality images and provide a food report—which is minimal, considering that only the meal name, time of intake, list of foods, and amounts eaten need to be texted—then confirm or adjust input as needed after review by the researcher. Although digital images usually include metadata on date and time the image was taken, we cannot assume that different mobile phone brands, models, and service carriers uniformly provide such information. Thus, we asked participants to still include the time their meals were eaten in their texted reports. Technology-assisted dietary assessment using computer algorithms to estimate food intake [30] holds great promise. However, fully automated analysis of digital images from mobile phones to identify and quantify foods could become inaccurate or lead to underestimation without additional information about the foods eaten [12]. For example, an automated image analysis that fails to distinguish a veggie burger from a meat burger would lead to inaccurate dietary analysis. Incorporating other technologies, such as voice recorders [31] and bar code scanners, may eventually improve the identification and quantification of food images. In the interim, human intervention is still necessary to supervise data collection, management, and analysis in ways that improve accuracy [13].

The incorporation of new dietary assessment technologies in mobile phone apps remains cost-prohibitive for use in large epidemiological studies. Although mobile phone food record apps are being developed and/or utilized, it will take time for the software algorithms to optimize human input. Until the full potential of these approaches are realized, our method of direct interpretation of simple texts and images can be considered feasible. However, we need to develop approaches that can speed up the transfer of texted food messages and digital images into files that can be easily encoded or linked with nutrient composition databases.

### Limitations

This study has a number of limitations. Although the method we used eased some of the burdens associated with pen-and-paper food recording, data collation, management, and quality control on the part of the researcher was time-consuming and prone to human error. However, feasibility studies on mobile phone food record apps developed to assess dietary intake of adolescents [17,20] have also demonstrated similar data management challenges. The lack of random assignment to the two intervention groups could have biased the results. The low-level intervention group had older participants, which might explain why the group had better results. However, cross-contamination between the two groups was also highly possible if the two interventions were simultaneously conducted. The two interventions were separated by 3-4 months which minimized possible cross-contamination. This arrangement also made the most logistical sense. Given that mobile phones with camera capability and unlimited phone service were necessary to conduct this study, this precluded participation of individuals who did not own mobile phones and/or high-quality phone service. Also, a 1-day food record may not provide enough information about adherence to instructions when food recording is done for more than 1 day. However, our intent in this study was to test the workability of self-reporting intake using the functions of personal mobile phones, so a 1-day food record would be considered sufficient. The email interface between participants and the researchers in the exchange of SMS text messages could be another limitation if technological glitches existed. However, we had tested this interface for different carriers and different mobile phones before the study started. Technological glitches, however, could possibly affect responsiveness of the high-level intervention group.

### Conclusions

Using personal mobile phones to report dietary intake via texting and taking digital images of foods eaten is feasible among free-living adolescents. High-level intervention involving real-time support is not a guarantee that the quality of food recording among adolescents will be more accurate. This food recording method is a prudent alternative until technology-assisted dietary assessment apps for mobile phones become widely available.

## Abbreviations

BMI: body mass index
EER: estimated energy requirement
EI: energy intake
FM: fiducial marker
FRDI: food record with digital images
ID: identification number
IQR: interquartile range
N/A: not applicable
NHANES: National Health and Nutrition Examination Survey
PA: physical activity
PAL: physical activity level
SMS: short message service

## Appendix

Multimedia Appendix 1 Sample of an actual 1-day food record with digital images from a study participant.

## References

[1] Bartholome LT, Peterson RE, Raatz SK, Raymond NC (2013). A comparison of the accuracy of self-reported intake with measured intake of a laboratory overeating episode in overweight and obese women with and without binge eating disorder. Eur J Nutr.

[2] Karelis AD, Lavoie M, Fontaine J, Messier V, Strychar I, Rabasa-Lhoret R, Doucet E (2010). Anthropometric, metabolic, dietary and psychosocial profiles of underreporters of energy intake: A doubly labeled water study among overweight/obese postmenopausal women--A Montreal Ottawa New Emerging Team study. Eur J Clin Nutr.

[3] Vance VA, Woodruff SJ, McCargar LJ, Husted J, Hanning RM (2009). Self-reported dietary energy intake of normal weight, overweight and obese adolescents. Public Health Nutr.

[4] Schap TE, Six BL, Delp EJ, Ebert DS, Kerr DA, Boushey CJ (2011). Adolescents in the United States can identify familiar foods at the time of consumption and when prompted with an image 14 h postprandial, but poorly estimate portions. Public Health Nutr.

[5] Boushey CJ, Kerr DA, Wright J, Lutes KD, Ebert DS, Delp EJ (2009). Use of technology in children's dietary assessment. Eur J Clin Nutr.

[6] Livingstone MB, Robson PJ, Wallace JM (2004). Issues in dietary intake assessment of children and adolescents. Br J Nutr.

[7] Alexy U, Wicher M, Kersting M (2010). Breakfast trends in children and adolescents: Frequency and quality. Public Health Nutr.

[8] Moreno LA, Rodriguez G, Fleta J, Bueno-Lozano M, Lazaro A, Bueno G (2010). Trends of dietary habits in adolescents. Crit Rev Food Sci Nutr.

[9] Goodwin RA, Brulé D, Junkins EA, Dubois S, Beer-Borst S (2001). Development of a food and activity record and a portion-size model booklet for use by 6- to 17-year olds: A review of focus-group testing. J Am Diet Assoc.

[10] Madden M, Lenhart A, Duggan M, Cortesi S, Gasser U (2013). Pew Research Center.

[11] Lenhart A (2015). Pew Research Center.

[12] Gemming L, Utter J, Ni Mhurchu C (2015). Image-assisted dietary assessment: A systematic review of the evidence. J Acad Nutr Diet.

[13] Martin CK, Nicklas T, Gunturk B, Correa JB, Allen HR, Champagne C (2014). Measuring food intake with digital photography. J Hum Nutr Diet.

[14] Storey KE (2015). A changing landscape: Web-based methods for dietary assessment in adolescents. Curr Opin Clin Nutr Metab Care.

[15] Stumbo PJ (2013). New technology in dietary assessment: A review of digital methods in improving food record accuracy. Proc Nutr Soc.

[16] Higgins JA, LaSalle AL, Zhaoxing P, Kasten MY, Bing KN, Ridzon SE, Witten TL (2009). Validation of photographic food records in children: Are pictures really worth a thousand words?. Eur J Clin Nutr.

[17] Daugherty BL, Schap TE, Ettienne-Gittens R, Zhu FM, Bosch M, Delp EJ, Ebert DS, Kerr DA, Boushey CJ (2012). Novel technologies for assessing dietary intake: Evaluating the usability of a mobile telephone food record among adults and adolescents. J Med Internet Res.

[18] Six BL, Schap TE, Zhu FM, Mariappan A, Bosch M, Delp EJ, Ebert DS, Kerr DA, Boushey CJ (2010). Evidence-based development of a mobile telephone food record. J Am Diet Assoc.

[19] Wang DH, Kogashiwa M, Ohta S, Kira S (2002). Validity and reliability of a dietary assessment method: The application of a digital camera with a mobile phone card attachment. J Nutr Sci Vitaminol (Tokyo).

[20] Casperson SL, Sieling J, Moon J, Johnson L, Roemmich JN, Whigham L (2015). A mobile phone food record app to digitally capture dietary intake for adolescents in a free-living environment: Usability study. JMIR Mhealth Uhealth.

[21] Buday R, Tapia R, Maze GR (2014). Technology-driven dietary assessment: A software developer's perspective. J Hum Nutr Diet.

[22] Schnall R, Okoniewski A, Tiase V, Low A, Rodriguez M, Kaplan S (2013). Using text messaging to assess adolescents' health information needs: An ecological momentary assessment. J Med Internet Res.

[23] Boushey CJ, Harray AJ, Kerr DA, Schap TE, Paterson S, Aflague T, Bosch RM, Ahmad Z, Delp EJ (2015). How willing are adolescents to record their dietary intake? The mobile food record. JMIR Mhealth Uhealth.

[24] IBM Analytics.

[25] Panel on Macronutrients, Panel on the Definition of Dietary Fiber, Subcommitee on Upper Reference Levels of Nutrients, Subcommittee on Interpretation and Uses of Dietary Reference Intakes, Standing Committee on the Scientific Evaluation of Dietary Reference Intakes, Food and Nutrition Board (2005). Dietary Reference Intakes for Energy, Carbohydrate, Fiber, Fat, Fatty Acids, Cholesterol, Protein, and Amino Acids.

[26] Murakami K, Livingstone MB (2016). Prevalence and characteristics of misreporting of energy intake in US children and adolescents: National Health and Nutrition Examination Survey (NHANES) 2003-2012. Br J Nutr.

[27] Rao S (1987). Variations in dietary intake of adolescents. Hum Nutr Clin Nutr.

[28] Maffeis C, Schutz Y, Fornari E, Marigliano M, Tomasselli F, Tommasi M, Chini V, Morandi A (2016). Bias in food intake reporting in children and adolescents with type 1 diabetes: The role of body size, age and gender. Pediatr Diabetes.

[29] Vance VA, Woodruff SJ, McCargar LJ, Husted J, Hanning RM (2009). Self-reported dietary energy intake of normal weight, overweight and obese adolescents. Public Health Nutr.

[30] Chen H, Jia W, Yue Y, Li Z, Sun Y, Fernstrom JD, Sun M (2013). Model-based measurement of food portion size for image-based dietary assessment using 3D/2D registration. Meas Sci Technol.

[31] Rollo ME, Ash S, Lyons-Wall P, Russell A (2011). Trial of a mobile phone method for recording dietary intake in adults with type 2 diabetes: Evaluation and implications for future applications. J Telemed Telecare.

