# Supplementary material for: Using Personal Mobile Phones to Assess Dietary Intake in Free-Living Adolescents: Comparison of Face-to-Face Versus Telephone Training
Source: JMIR Mhealth Uhealth. 2016 Jul 29;4(3):e91. doi: 10.2196/mhealth.5418 (PMC4982913; doi:10.2196/mhealth.5418)

ID# Name

Report Date: mm/dd/yyyy, Day

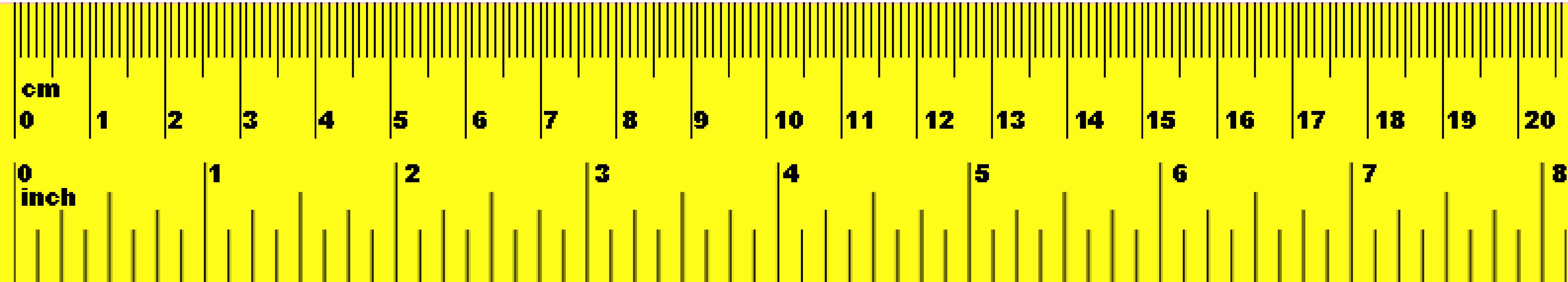

B-fast (Before)

Time: 7:35am

Description:

- 3 morning star vege patties
- 1 croissant
- 1 kiwi
- 7 Oz strawberry yogurt

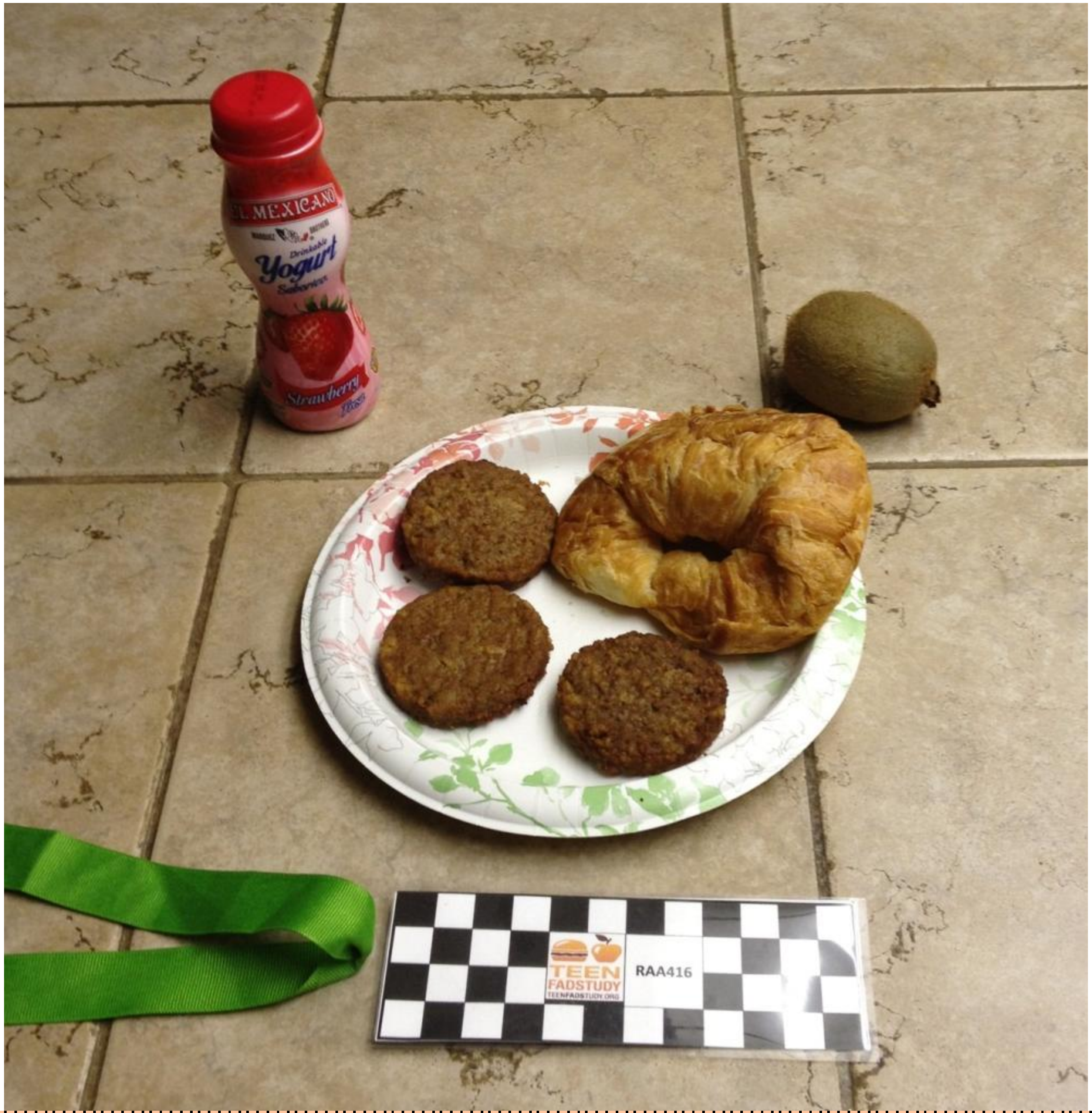

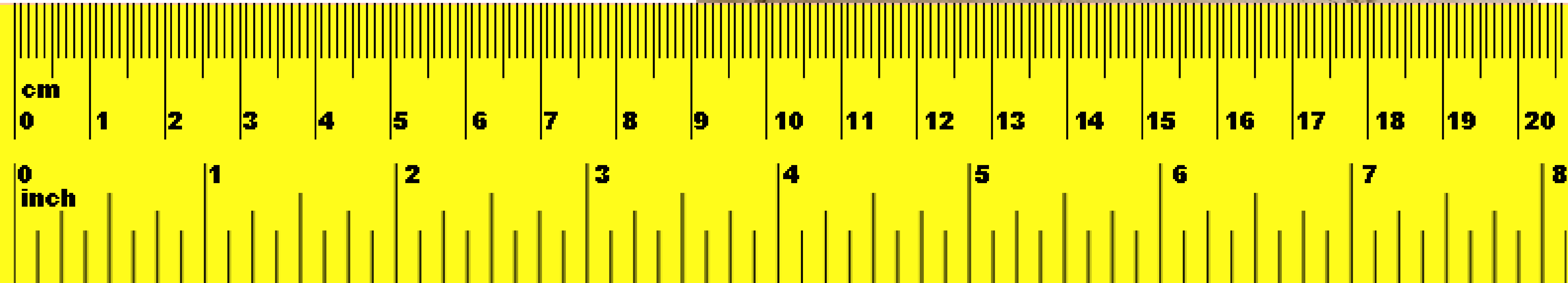

Lunch-Before

Time: 11:47am

Description:

- 1 vege hotdog with 1 tsp mustard, 1 tsp ketchup, 1 tsp pickle relish
- 1.5 cups of baked French fries
- 1 cup cold chili
- 1/4 an apple
- 1 slice cucumber
- 3 baby carrots
- 1 baby tomato
- 8th continent soymilk a half pint

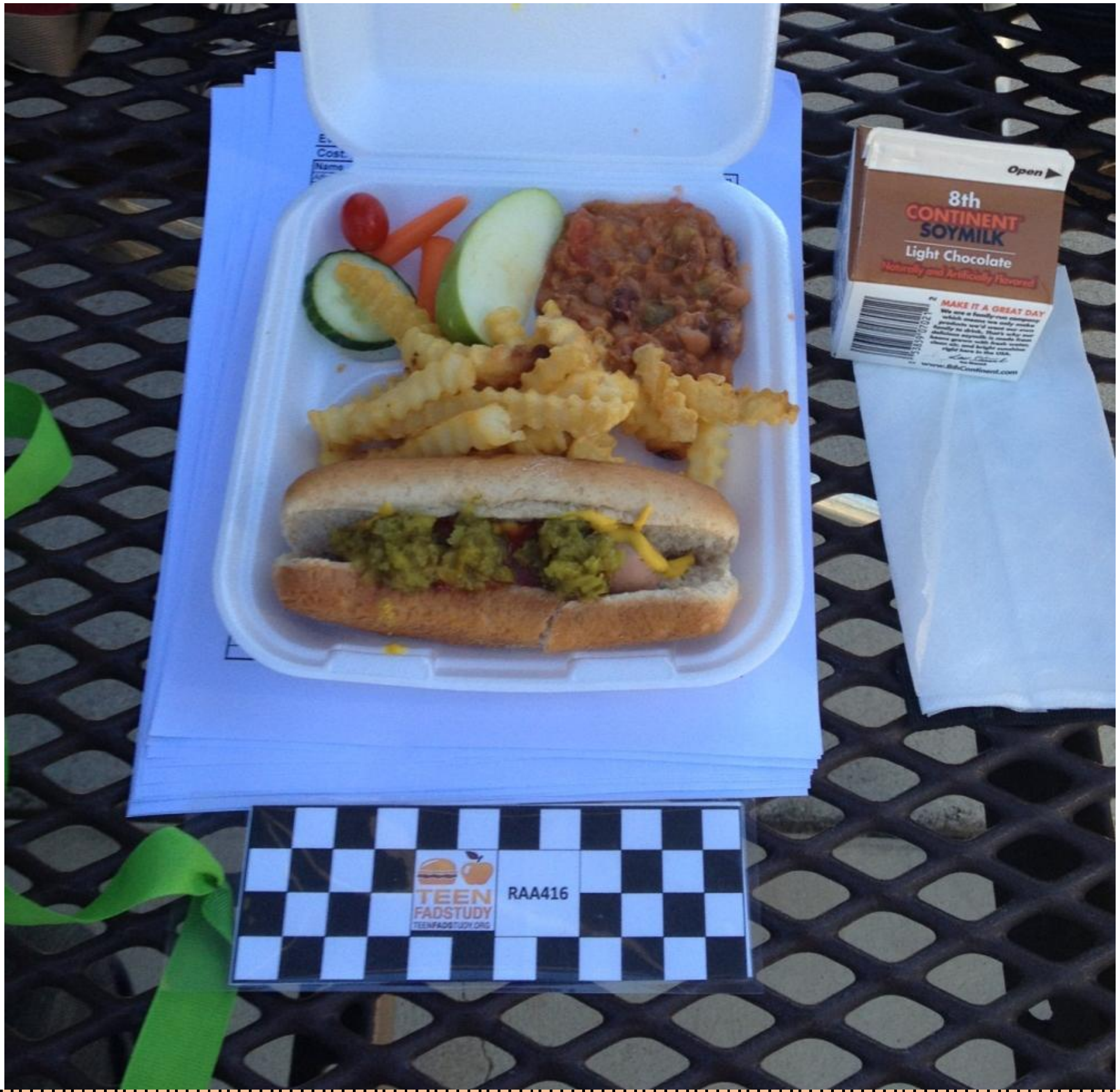

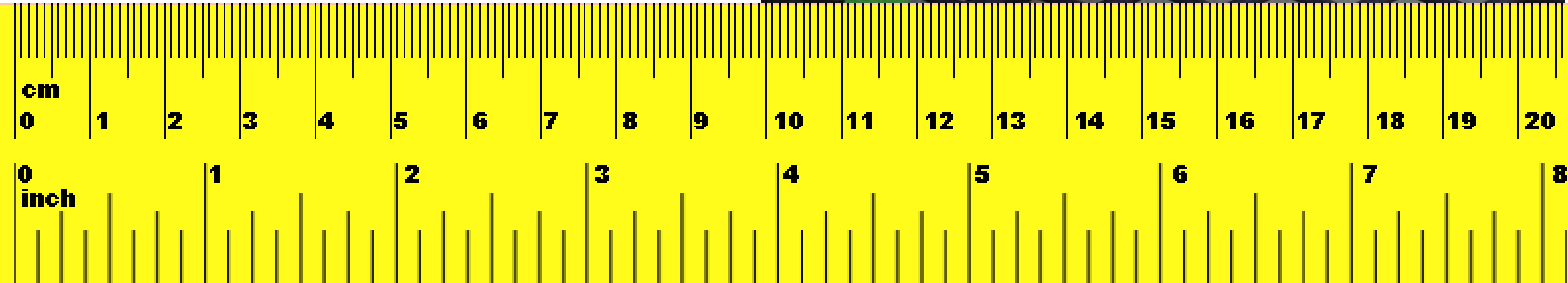

Dinner-before

Time: 8:33pm

Description:

-1 cup of corn

-1 cup of green beans

-1.5 cup of white rice

-1 slice of chicken breast with ¾ cup of cream of mushroom and paprika sauce

-1 cup of water

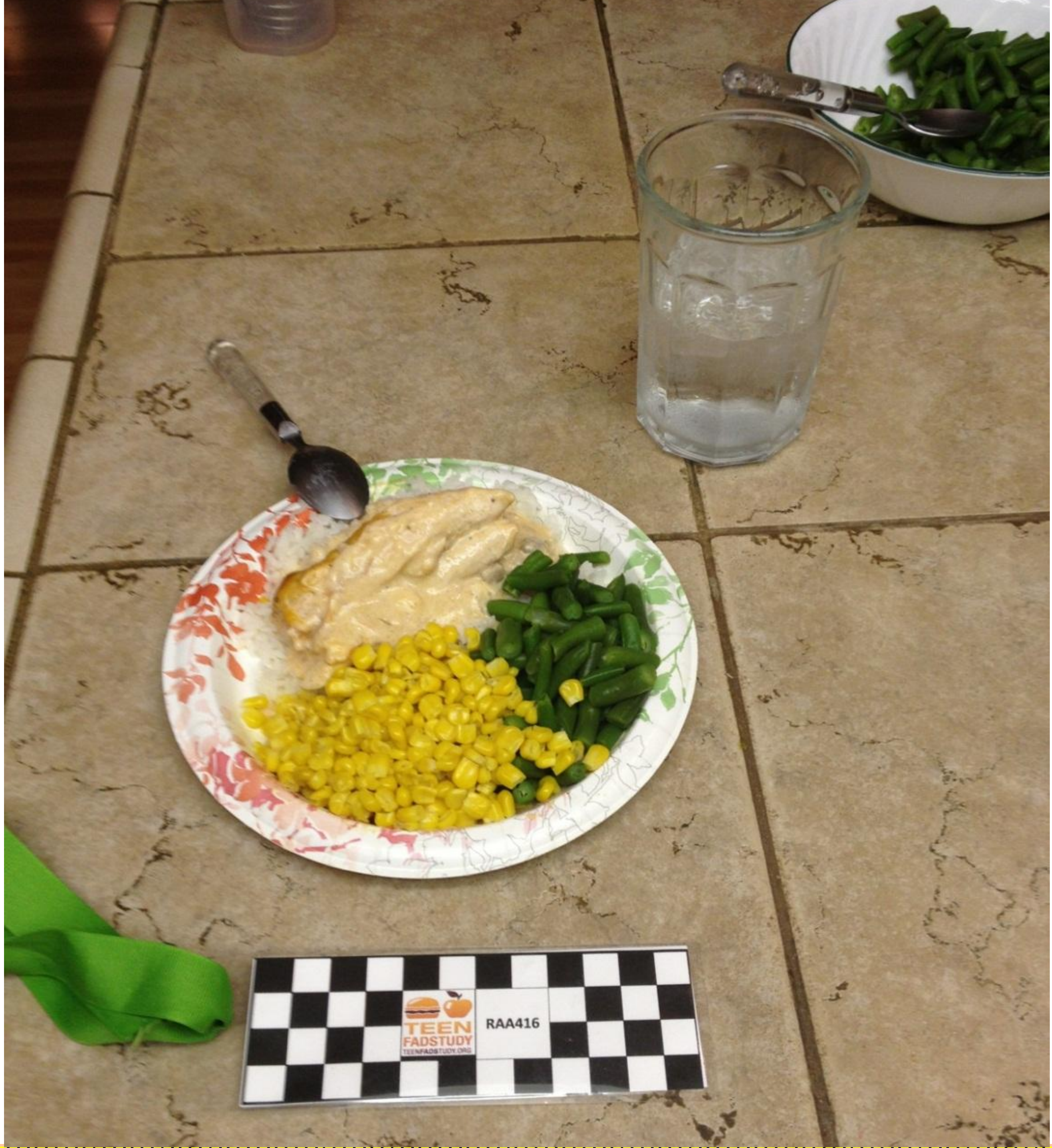

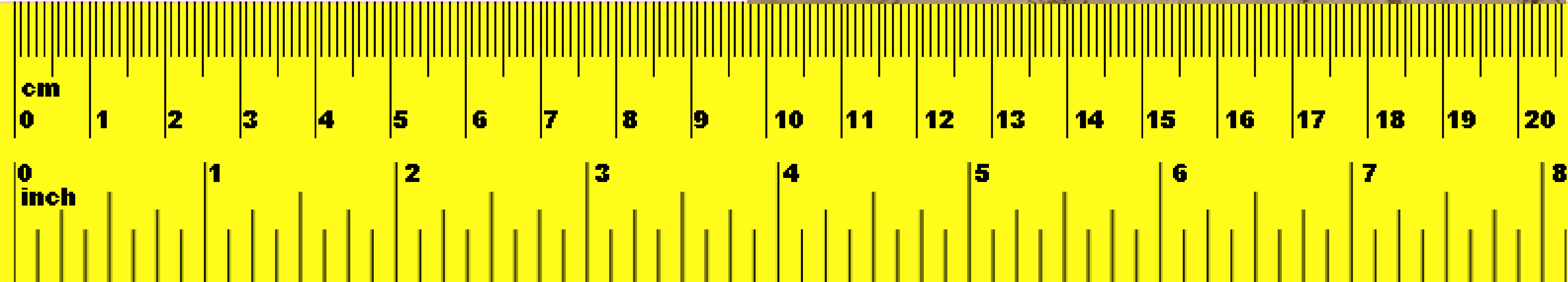

End- 10:36pm

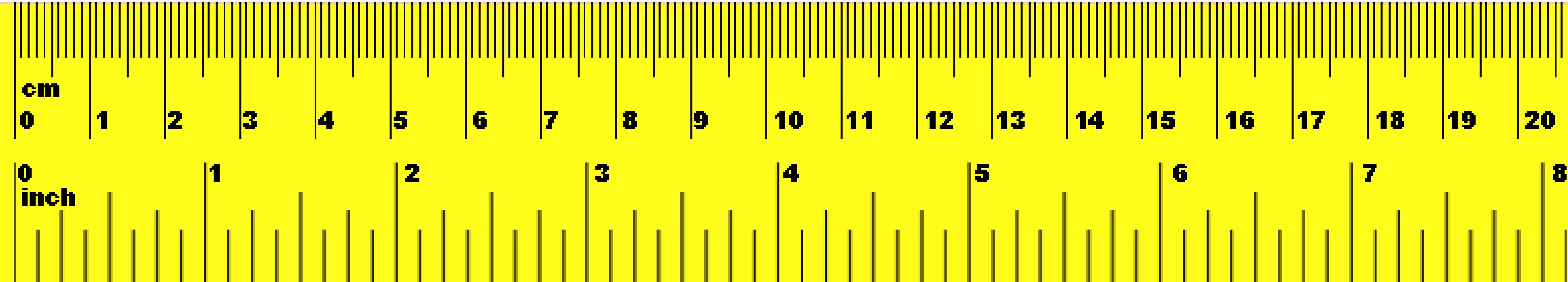

Supplement: Multimedia Appendix 1 [file mhealth_v4i3e91_app1.pdf]
